# The Lived Experience of Assisted Living Administrators During the COVID-19 Pandemic

**DOI:** 10.1093/geroni/igab046.2712

**Published:** 2021-12-17

**Authors:** Elizabeth Hill, Rebecca Davis, Paige Greer, Susan Strouse

**Affiliations:** Grand Valley State University, Grand Rapids, Michigan, United States

## Abstract

Since March, 2020, administrators in assisted living (AL) residences have been challenged to provide the best care for their populations while undergoing a pandemic. Because nothing like this has happened in the recent past, AL administrators had to make many new decisions. The purpose of this phenomenological study is to reflect on the lived experiences of AL administrators during the COVID pandemic. Using a semi structured interview, individual interviews of four AL administrators from different AL communities were conducted via Zoom. The interviews contained questions related to the participants’ experiences with the COVID-19 pandemic. The recorded interviews were transcribed verbatim into MAXQDA. Data analysis followed a modified Giorgi approach, by reviewing the recorded interviews, categorizing the data into meaning units, then situated units, generalized units, and then themes. Results indicated that AL administrators have been adapting to constantly changing and conflicting regulations. The pandemic incited fear, depression, moral distress, but also hope for the future. The data shows a multitude of feelings and actions related to the well-being of the staff, residents, and residents’ families. The limitations of this study include a small sample size and the evolving nature of the pandemic in Michigan. Opportunities for future research would be to compare our findings to the experience of other AL’s in the United States. The results show the complexity of AL administrators’ lived experiences during the pandemic and highlight important considerations if an event like the pandemic were to occur again.

